# Supplementary material for: SyNDI: synchronous network data integration framework
Source: BMC Bioinformatics. 2018 Nov 6;19:403. doi: 10.1186/s12859-018-2426-5 (PMC6219086; doi:10.1186/s12859-018-2426-5)
Supplement: Supplementary file 5 — Files of the example presented in Additional file 4. This additional file is a zip package that contains all files used in Additional file 4. Detailed descriptions of each of these files are presented in a README file included in this zip package. (GZ 30411 kb) [file 12859_2018_2426_MOESM5_ESM.gz › SyNDITools/Meme2Fimo/meme_4.9.0_4.tar.gz!/meme_4.9.0/doc/pmp_bf.xml]

pmp\_bf


# pmp\_bf

**Usage:** `pmp_bf [options] <tree> <motif>`

**Description:**

This utility calculates the statistical power of a phylogenetic motif model.

**Input:**

- <*tree*> - a file containing a phylogenetic tree in
  Newick format.
- <*motif*> - a file containing a set of motifs in
  MEME format.

**Output:**

The program prints to standard output.

**Options:**


**Bugs:** None known.
